# A systematic review of functional neurological disorder in transgender people

**DOI:** 10.1080/26895269.2025.2538742

**Published:** 2025-07-25

**Authors:** Richard C. J. Bradlow, Sav Zwickl, Patrick Reardon, Ada S. Cheung, Richard A. Kanaan

**Affiliations:** a Department of Psychiatry, University of Melbourne, Austin Health, Heidelberg, Australia; b Trans Health Research Group, Department of Medicine, The University of Melbourne, Heidelberg, Australia

**Keywords:** Functional neurological disorders, transgender persons, Gender affirming hormone therapy (GAHT), Somatic symptom disorder, Gender minority stress

## Abstract

**Background:**

Trans and gender diverse people may be more likely to have a functional neurological disorder (FND), although the reasons for this are unclear. This systematic review examines the relationship between trans people and FND, to determine if one population is overrepresented in the other, and if gender affirming-care affects symptoms of FND.

**Methods:**

A systematic review was conducted as per PRISMA guidelines. Searches were performed on OVID (MEDLINE), PsycINFO, and Web of Science. Studies included focused on both FND and trans people, had subjects of any age, and were original research articles. When the study referred to somatic symptom and related disorders it was included. Study quality was assessed using JBI’s critical appraisal tools.

**Results:**

Fourteen studies were included. Seven examined the prevalence of FND (or likely FND) in trans people, or of trans people in people in FND or (likely FND patient populations), and seven were case studies or series of trans people with probable FND (total cases = 20). Trans people made up 3–45% of people with FND; somatic symptom and related disorders were present in 6–9.7% of trans people (compared to 5–7% in the general population). Fifteen of the 20 cases were trans people assigned female at birth. Ten of the 20 cases developed FND prior to their social gender affirmation, and seven of those ten’s symptoms resolved with gender affirmation.

**Discussion:**

Trans people were more likely to develop FND, and somatic symptom and related disorders were slightly overrepresented in trans people. Gender affirmation can lead to resolution of FND symptoms. Trans people appear to make up a considerable proportion of people with FND. FND treatment services need to consider how they provide trans affirming services.

## Introduction

Functional neurological disorder (FND) (a somatic symptom and related disorder) is an illness characterized by altered motor or sensory function that causes substantial distress, and is not found to have a neurological, medical, or psychiatric explanation (American Psychiatric Association, 2013). The prevalence rate is approximately 0.05% (Aybek & Perez, 2022) and FND is the most common diagnosis seen in neurology clinics in Australia (Ahmad & Ahmad, 2016). Symptoms can be wide ranging including myoclonus, seizures, and cognitive impairment. People who have FND experience significant functional impairment, distress, stigma, and reduced quality of life (Carson et al., 2011; Tinazzi et al., 2021). Trans people are at higher risk of multiple negative physical and mental health outcomes compared to their cisgender counterparts (Boyer et al., 2021; Hughes et al., 2022; Rich et al., 2020), and trans people have previously been found to be overrepresented in FND (Bradlow et al., 2024), for reasons that are unclear. Trans people with FND can be stigmatized for both their gender modality and their illness, putting them in a highly vulnerable position.

Whilst psychosocial factors (e.g., trauma, social influences, or physical injury) play a major role in the pathogenesis of FND, there may also be biological contributors (Baizabal-Carvallo et al., 2019). There appears to be a marked difference in the gender ratio in FND, with current estimates purporting that approximately three quarters of people with FND are women (Lidstone et al., 2022). It is unclear why there is such a marked gender ratio, however, it has been hypothesized that psychosocial factors (e.g., associated discrimination and gender power imbalance) (McLoughlin et al., 2023) and sex hormones (estrogen, progesterone, oxytocin, and endogenous opioids) may both play a role in the pathogenesis of FND (Chouksey & Pandey, 2020).

A limitation of current research is the conflation of sex assigned at birth and gender, such that it is unclear how trans people are categorized in these samples, if included at all. For example, it is unclear across much of the research whether ‘women’ refers to persons who identify as women (cisgender and trans women), or persons assigned female at birth (cisgender women and trans people assigned female at birth). Further, sex hormone levels have not previously been reported in FND nor have the effects of exogenous hormones (gender affirming hormone therapy (GAHT)) on trans people with FND.

The gender minority stress model proposes that the lower the prevalence and more marginalized a gender modality is, the higher the trauma burden and the worse their social and psychiatric outcomes will be (Pace et al., 2021). It may be expected that FND would be more prevalent in trans people compared to cisgender people secondary to substantial barriers to mental health and wellbeing, including stigma, discrimination and difficulty accessing healthcare. FND may be expected to occur less frequently and improve with all gender affirmation due to improved quality of life and reduced psychiatric burden. However, if sex hormones play a role in the pathogenesis of FND, GAHT specifically may be associated with a higher likelihood of developing FND, or for FND to be less likely to resolve after GAHT.

This systematic review aims to examine the literature to assess the relationship between trans people and FND. Specifically, it aims to assess if trans people are overrepresented in FND, if FND is more prevalent in trans people compared to cisgender people, and if gender affirming care provokes or improves the symptoms of FND.

## Methods

Searches were performed on OVID (MEDLINE), PsycINFO, and Web of Science. Full search terms can be accessed in the supplemental material (see Table S1 in Supplementary Materials). A secondary search was conducted on Google Scholar and the bibliographies of all papers that were reviewed at full text were also manually searched for relevant papers. The searches were conducted on 8 April 2024 and updated on 24 June 2025.

Papers were screened using the Covidence web tool. All papers were screened by title and abstract independently by RB (psychiatrist) and PR (medical student) with disputes resolved by RK (psychiatrist). Full texts were also reviewed independently by RB and PR with disputes resolved by RK.

The review inclusion criteria were: study focus on both FND and trans people; subjects were human of any age; and study design was a randomized controlled trial, an observational study, or a case report or series. Some studies used the diagnosis “somatoform disorder”, which corresponds with “somatic symptom and related disorders” in the DSM 5 (American Psychiatric Association, 2022). Somatoform disorders/somatic symptom and related disorders encompasses multiple diagnoses including FND. When a study referred to a diagnosis that could be FND, such as “somatoform disorder”, without specifying further, it was included and put forward to the next stage, or ultimately included with appropriate caveats if at the full-text stage.

Data was first extracted on 15 April 2024. Data was extracted by RB and PR. Data was synthesized and grouped thematically (by population-based studies and case reports and series) by RB. Case data were presented in tables. Papers were reviewed for the prevalence of FND in trans people and the prevalence of trans people in FND. Individual cases were reviewed for the timing and course of FND in relation to gender affirmation. Meta-analysis was considered but not possible due to small numbers and interstudy heterogeneity.

When indicated, attempts were made to contact authors of other papers for clarification.

Study quality was assessed using JBI’s critical appraisal tools by RB and PR (Joanna Briggs Institute, 2023).

## Results

A total of 553 articles were identified through the database search. A further 3 articles were identified *via* searching citations. One hundred duplicates were removed, 428 studies were excluded at screening stage due to irrelevancy, 14 studies were excluded on full text screen (4 had the wrong design, 9 the wrong patient population, and one was secondary analysis). A total of 14 articles were included in the review (see Figure 1).

**Figure 1.**
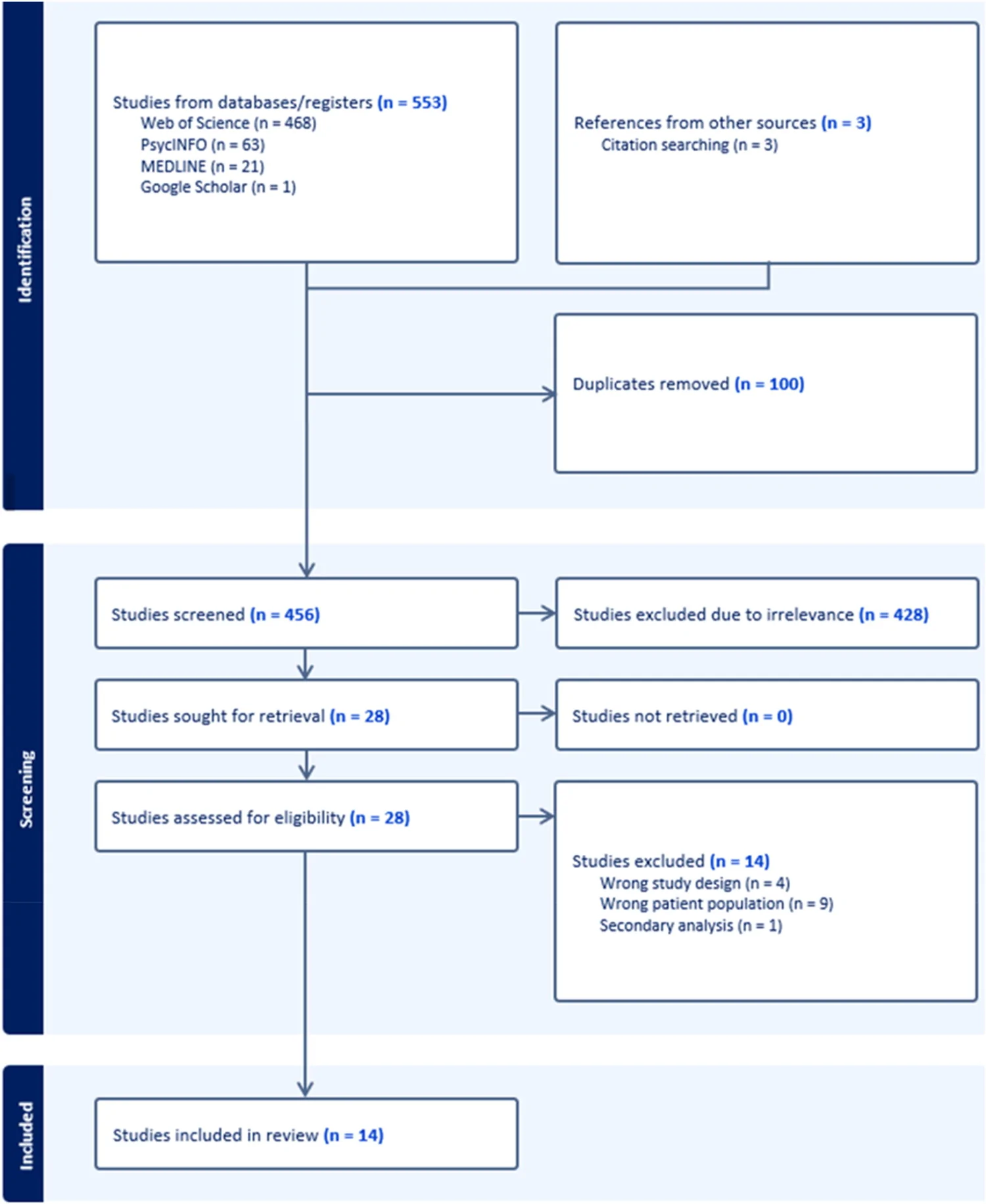
PRISMA flow diagram.

### Study characteristics

Of the 14 studies included for review, seven studies (three cross sectional, two retrospective cohort, and two case control studies) examined the prevalence of one population (trans or FND) in the other and seven were case studies or series. A majority of the case studies/series (five of seven) were of adolescents.

### Studies on the prevalence of FND in trans people and trans people in FND

There were five studies that looked explicitly at the prevalence of trans people in FND. A retrospective cohort study (*n* = 154) of an Australian FND clinic from 2014 to 2019 found that the prevalence of trans people was 3% (Morsy et al., 2021). A follow up case series from the same clinic (2014–2022) found similar prevalence (3.2% of 282 patients) (Bradlow et al., 2024).

One study reported on the prevalence of non-binary people in a descriptive study of mass sociogenic illness (social media content creators with functional tic disorders). It found that 14.3% (*n* = 4) of 28 content creators with functional tic disorder were nonbinary (Olvera et al., 2021). However, it is not clear whether the other 81.9% (24 people) who reported their gender as male or female were cisgender or trans (Olvera et al., 2021).

A cross-sectional case control study compared patients with functional tics recruited from a pediatric movement disorder clinic with matched Tourette’s syndrome and neurotypical controls, found that 42.8% of the participants (*n* = 15) with functional tics were trans (Berg et al., 2024), significantly higher than in both control groups. Similarly, a retrospective cohort study of new onset functional tic presentations to a pediatric hospital found that 45% of the participants (*n* = 25) were trans (Tomczak et al., 2024). Another cross-sectional study of children and adolescents with functional tics seen in a movement disorder clinic found that 10.5% of participants who reported their gender as female were trans (*n* = 2) (Armstrong-Javors et al., 2024). Thirty-seven percent of participants were reported as having either gender or sexual diversity, without further specifying, and thus it is possible that more than two of the participants were transgender.

There were no studies on the prevalence of FND, specifically, in trans people. However, two studies reported on the presence of somatoform disorders (which includes FND) in trans people. A large (*n* = 1605), retrospective, case-control study of a German database of private patients in general and specialist practices found that somatoform disorders were present in 9.7% of trans people, which was significantly higher than their cisgender matched controls (5.8% in both cisgender men and cisgender women) (Konrad & Kostev, 2020).

A small (*n* = 50) cross-sectional study of the Hijra community, a cultural and social group in India and Pakistan who are recognized as a "third gender", generally assigned male at birth or with an innate variation of sex characteristics but have a female gender or gender other than male or female, found that 6% had a history of a somatoform disorder (Sartaj et al., 2021).

### Case reports and series

The five case reports and two case series included ten published cases of trans adolescents developing a somatic symptom and related disorder (see Table 1) and two cases of trans adults with FND (one as an illustrative case in a case series).

**Table 1.** Population studies.

Study	Country	Setting	Design	Sample size	Diagnosis	Percentage of participants trans	Percentage of participants with diagnosis	Notes
Konrad and Kostev (2020)	Germany	Primary healthcare	Retrospective case-control	1605	Somatoform disorders	NA	9.3%	Compared to 5.8% in cisgender men and 5.8% in cisgender women
Sartaj et al. (2021)	India	Hijra community	Cross-sectional	50	Somatoform disorders	NA	60025	
Morsy et al. (2021)	Australia	FND clinic	Retrospective cohort	154	FND	3%	N/A	2014–2019; prevalence of trans people in FND
Olvera et al. (2021)	Unknown	Social media	Descriptive study	28	FND (functional tic disorders)	14.3% (non-binary)	NA	Unclear gender modality of other 24 participants
Bradlow et al. (2024)	Australia	FND clinic	Retrospective cohort	282	FND	3.2%	N/A	2014–2022; percentage trans people in FND, same clinic as Morsy et al. (2021)
Berg et al. (2024)	United States of America	Pediatric movement disorder clinic	Cross-sectional case control	35 (with a functional tic disorder)	FND (functional tic disorders)	42.8 %	NA	Compared to 9.1% of participants with Tourette’s syndrome and 12% of neurotypical participants
Tomczak et al. (2024)	United States of America	Pediatric hospital	Retrospective cohort	56	FND (functional tic disorders)	45%	NA	
Armstrong-Javors et al. (2024)	United States of America	Pediatric movement disorder clinic	Cross sectional	19	FND (functional tic disorders)	10% transgender	NA	37% were of sexual or gender minority, it is unclear how many were trans

**Table 2.** Cases.

Study	Gender	Age at onset of symptoms	Sex assigned at birth	Symptom onset prior to gender affirmation	Symptoms	Diagnosis assigned	Co-occurring mental illness	Effect of gender affirmation on Somatic Symptoms
Orfanelli and Borkowski (2006)	Male	10	Female	Yes	Myotonic jerking, repetitive guttural noises, episodic episodes of dizziness	Conversion disorder	ADHD, GAD, PANDAS, chronic motor tics, Tourette syndrome, OCD	Symptoms resolved following social affirmation as a trans man
Schif et al. (2010)	Not reported	14	Male	Yes	Unilateral paralysis, sensory loss and diminished reflexes	Psychogenic hemiplegia	Not discussed	Symptoms resolved with “emotional treatment”, not discussed in the paper if they affirmed their gender
Williams (2012)	Female	62	Male	No	Quadriplegia, difficulty urinating	Spinal cord compression with overlay of functional symptoms	Histrionic personality traits, alcohol and cocaine dependence	Ongoing symptoms at the time of publishing
Boris et al. (2019)	Male	15	Female	Yes	Dizziness, headaches, fatigue, cognitive dysfunction, insomnia, early satiety, numbness, joint pain, heat and cold intolerance, exercise intolerance, dyspnea, diaphoresis, palpitations, tachycardia, photophobia, hyperacusis	POTS	None	Received GAHT (testosterone) aged 17. All symptoms, except joint pain, resolved by age 20. GAHT improved symptoms.
Boris et al. (2019)	Male	12, symptoms onset with menarche	Female	Yes	Dizziness, blurry vision, syncope, fatigue, cognitive dysfunction (improved with atomoxetine), insomnia, headache, nausea, diarrhea, constipation, early satiety, abdominal pain, numbness, muscle pain, joint pain, heat intolerance, dyspnea with activity, chest pain, palpitations, tachycardia, photophobia, hyperacusis	POTS	ADHD, BPAD, OCD	Received GAHT (testosterone) aged 19. GAHT improved symptoms.
Boris et al. (2019)	Male	6	Female	Yes	Migraine, dizziness, blurred vision, fatigue, cognitive dysfunction, insomnia, nausea, early satiety, abdominal pain, numbness, muscle pain, joint pain, heat and cold intolerance, exercise intolerance, dyspnea with activity, diaphoresis, chest pain, palpitations, tachycardia, photophobia, hyperacusis	POTS	Anxiety disorder, OCD, depression	Received GAHT (testosterone) aged 18. GAHT improved dizziness, insomnia, fatigue, headache, and cognitive dysfunction. Symptoms returned during gaps in his testosterone therapy
Morabito et al. (2021)	Male	15	Female	Yes	Arthralgia, myalgia, headache, and fatigue	Somatic symptom disorder	Not discussed	Did not receive GAHT. however, symptoms improved following social affirmation as a trans man.
Wilkinson-Smith et al. (2023)	Male	15	Female	No	Gait difficulties, forced eye closure	FND	Depression, anxiety	Received GAHT (medroxyprogesterone and testosterone injections) aged 13, prior to the onset of FND and GAS (chest reconstruction surgery) aged 16. Symptoms largely resolved by 17
Wilkinson-Smith et al. (2023)	Non-binary	11	Male	No	Gait difficulties, weakness, tremor	FND	Depression, anxiety	Received GAHT (puberty blockers). Symptoms improved with typical FND management, no apparent effect of GAHT on FND
Wilkinson-Smith et al. (2023)	Gender fluid	14	Female	No	Gait difficulties, tremor	FND	Anxiety	Did not receive GAHT. Unknown outcome as lost to follow up
Wilkinson-Smith et al. (2023)	Male	16	Female	Yes	Tics, gait difficulties, loss of consciousness	FND	Anxiety with panic attacks	Received GAHT (testosterone) aged 17. Tics and loss of consciousness improved after affirming their gender socially but gait difficulties subsequently developed. GAHT resulted in improvement in all symptoms.
Bradlow et al., 2024)	Non-binary	28	Female	No	PNES, reduced energy (emotional, physical and mental), cognitive symptoms (difficulties multitasking and with memory)	FND	PTSD, depression, anxiety	Received GAS (chest reconstruction surgery) which had no apparent effect on FND

ADHD = attention deficit hyperactivity disorder, GAD = generalized anxiety disorder, PANDAS = pediatric autoimmune neuropsychiatric disorders associated with streptococcal infections, OCD = obsessive compulsive disorder, POTS = postural orthostatic hypertension syndrome, GAHT = gender affirming hormone therapy, BPAD = bipolar affective disorder, GAS = gender affirming surgery, FND = functional neurological disorder, PNES = psychogenic non-epileptic seizures, PTSD = post-traumatic stress disorder

Six of the ten adolescent cases were diagnosed with FND (Orfanelli & Borkowski, 2006; Schif et al., 2010; Wilkinson-Smith et al., 2023), one with somatic symptom disorder (Morabito et al., 2021), and three with postural orthostatic tachycardia syndrome (POTS) (Boris et al., 2019), a condition which it has been argued is FND (Norcliffe-Kaufmann et al., 2022). However, all cases were described with multiple symptoms consistent with FND, even when not labeled as such by the authors.

Of the ten adolescent cases, eight were assigned female at birth, six of whom were likely post pubescent at the onset of their FND symptoms, given their ages (12–16) and thus had more circulating estradiol than the cases aged 10 and 6 (Boris et al., 2019; Morabito et al., 2021; Orfanelli & Borkowski, 2006; Schif et al., 2010; Wilkinson-Smith et al., 2023). However, one case was also receiving medroxyprogesterone and testosterone at the onset of their symptoms (Wilkinson-Smith et al., 2023). At the time of onset of their symptoms, five cases were trans women, two were trans men, two were non-binary and one was gender fluid. Two had an improvement in symptoms following social affirmation, four with GAHT (testosterone therapy), one with gender affirming surgery (GAS), and in one case the effect of GAHT (puberty blocker therapy) appeared to be minimal or was unknown (Boris et al., 2019; Morabito et al., 2021; Orfanelli & Borkowski, 2006; Wilkinson-Smith et al., 2023).

The case report of a trans adult with FND was of a trans woman who had a spinal pathology with functional overlay (Williams, 2012). She had previously had GAS, but it was not reported if she had any history of GAHT. Of note is that she was initially diagnosed with FND alone and her organic injuries were missed, leading to delayed treatment. The case series of trans adults with FND (discussed in the previous section too) only presented one case in detail, presenting the others in aggregate. Seven of the nine cases were assigned female at birth, this included three trans men, two non-binary people, and two individuals had not disclosed they were trans at the time of diagnosis. The remaining two were trans women (Bradlow et al., 2024). FND symptoms began before social affirmation in three of the nine cases. The effect of being able to affirm one’s gender on FND in these adult cases was unknown, except in one case, where no effect was (Bradlow et al., 2024).

Overall, 15/20 cases were assigned female at birth, 10/20 had socially affirmed their gender prior to the onset of their FND, and 7/10 people whose FND symptoms emerged before gender affirmation, had a resolution of symptoms with gender affirmation.

## Study quality

Several studies included have a high risk of selection bias; Armstrong-Javors et al., Berg et al., Bradlow et al., and Morsy et al. all recruited from tertiary clinics (Armstrong-Javors et al., 2024; Berg et al., 2024; Bradlow et al., 2024; Morsy et al., 2021). Olvera et al. also has a high risk of selection bias as they reported on social media users which are unlikely to be representative (Olvera et al., 2021). Likewise, several case reports (Morabito et al., 2021; Orfanelli & Borkowski, 2006; Schif et al., 2010; Williams, 2012) were reported due to complex presentation or outcome and thus have a high risk of selection bias.

Morsy et al. and Bradlow et al. had moderate risk of bias from varied follow-up in retrospective cohort studies, in which some patients would have had follow-up of up to five years and others of a few months (Bradlow et al., 2024; Morsy et al., 2021). Some patients may have been lost to the clinic beyond their initial appointments and, may have not disclosed to the clinic they were trans or affirmed a gender different to the gender socially attributed to the sex assigned to them at birth following their period of being known to the clinic (Bradlow et al., 2024; Morsy et al., 2021). Likewise, both papers employed retrospective analysis of notes and thus progress in both gender and FND in many patients was unknown (Bradlow et al., 2024).

There was a moderate risk of bias in analyzing gender in Olvera et al., as they reported identified gender and did not report on whether participants were trans (Olvera et al., 2021). Armstrong-Javors et al. didn’t report in detail the gender and sexual diversity present in participants, or report the exact number of participants who were trans (Armstrong-Javors et al., 2024).

Schif et al. did not clearly describe their patients’ gender dysphoria nor adequately describe the course of their gender after presentation (Schif et al., 2010). One case in Wilkinson-Smith et al. was lost to follow-up and thus their progress in gender, gender affirmation and FND is unknown (Wilkinson-Smith et al., 2023).

## Discussion

This review examined seven published population studies, and seven case series or reports of trans people with FND. It found that trans people were over represented in FND compared to the general population (3–45% in FND and 0.5–4.5% in the general population) (Bradlow et al., 2024; Collin et al., 2016; Morsy et al., 2021; Spizzirri et al., 2021; Zhang et al., 2020). It also found that somatoform disorders (but not necessarily FND) appeared modestly more prevalent in trans people compared to cisgender people (6–9.7% in trans people and 5–7% in the general population) (Konrad & Kostev, 2020; Sartaj et al., 2021). Though these population differences were not statistically compared in most studies, the difference was more pronounced and statistically significant in the one study that did (Konrad & Kostev, 2020).

The onset of FND symptoms occurred prior to social affirmation in 10/20 cases. Gender affirmation was associated with an improvement in FND symptoms in 7 of the 10 cases in which FND symptoms emerged prior to social affirmation and was not reported to be associated with a deterioration in symptoms in any cases. This indicates that gender affirmation may assist in recovery from FND and that screening for gender dysphoria should be integrated into assessments of FND. It is notable that all seven cases that improved following gender affirmation were adolescent cases. There was insufficient evidence in the reviewed studies to state if any aspect of gender affirmation had a more pronounced effect on FND symptoms; social affirmation was documented as having a positive effect on somatic symptoms in two cases, GAS as having a positive effect on symptoms of FND in one case, and GAHT in four cases. These findings highlight the profound positive effect that gender affirmation can have and is in keeping with existing literature (van Leerdam et al., 2023). There was insufficient evidence to determine whether there was a difference in the effect of different GAHT on FND symptoms, however it is notable that of the six published cases that received GAHT, four had an improvement of symptoms post-GAHT that the authors attributed to GAHT. All four of those cases were trans men receiving testosterone therapy.

The studies of functional movement disorder clinics post the onset of the COVID-19 pandemic reported a much higher prevalence of trans participants than the other studies (10–45% v 3–3.2%) (Armstrong-Javors et al., 2024; Berg et al., 2024; Bradlow et al., 2024; Morsy et al., 2021; Tomczak et al., 2024). This is in keeping with existing literature that the pandemic led to disruptions to gender affirming care and an increase in depression amongst trans people (Zwickl et al., 2024, 2025). Borderline personality disorder, agoraphobia, social anxiety disorder, and generalized anxiety disorder were associated with an increased risk of developing a functional movement disorder during COVID-19 (Berg et al., 2024). The latter three have been found to be overrepresented in trans people (Millet et al., 2017).

### Significance of findings

That trans people make up 3–45% of people with FND is striking, as it means they are a sizable portion of the FND patient population. For context, 5% of FND patients present with jerks/myoclonus (Lidstone et al., 2022) yet there has been a substantial body of research published on the topic. Somatoform disorders being more common in trans people is in keeping with the literature in related disorders: an online survey of trans people that found a high prevalence of somatization symptoms (27.5%) measured on the Brief Symptom Inventory (Bockting et al., 2013), a cross-sectional study of trans people that found that somatoform disorders were present in three (9.7%) patients (Hepp et al., 2005), and that somatization has been found to be higher in trans people as measured on the Minnesota multiphasic personality inventory (Gherman et al., 2022; Tsushima & Wedding, 1979).

It appears that being able to affirm one’s gender may assist with the resolution of FND symptoms; however, it is not clear if this is secondary to benefits to mental health and wellbeing from gender affirmation, or from the hormonal effects of GAHT. However, at this stage there is insufficient evidence to clinically recommend gender affirmation solely for the management of FND in trans people. It may be that the beneficial effects of gender affirmation on FND are more pronounced in adolescents. Given that social affirmation improved FND in two cases, it is evident that, in some cases, alleviating a degree of mental distress from gender affirmation can improve FND symptoms.

The psychosocial factors that contribute to gender differences seen in FND such as under-recognition of FND in cisgender men and risk factors that, due to societal inequity, disproportionately affect women: sexual abuse, violence, and political and socioeconomic disparities (McLoughlin et al., 2023) are present and possibly heightened in trans people who experience considerable discrimination and stigma that could explain their overrepresentation in FND (McLoughlin et al., 2023). This increased trauma burden in trans people is believed to be due to higher societal discrimination as outlined in the gender minority stress model (Bradlow et al., 2024). The overrepresentation could also be secondary to confounding factors such as experiences of trauma or autism, both of which have a higher prevalence in trans people compared to the general population (Bradlow et al., 2024; Cheung et al., 2018; González-Herrero et al., 2022; Rosendale et al., 2021). Possible explanations for autism being overrepresented in gender diverse people are that autism may be over diagnosed in trans people (Cheung et al., 2018; Rosendale et al., 2021), that autistic people may feel less need to conform societal norms, thus making them more likely to express gender identification outside of the binary (Warrier et al., 2020), or that they may share a precursor (e.g., that prenatal influences on brain development contribute to a person being more likely to neuroatypical and trans) (Warrier et al., 2020). Autism appears to be overrepresented in FND perhaps because it is associated with altered attention circuitry, alexithymia, an altered sense of agency, and higher sensory sensitivity, all of which can play a role in the pathogenesis of FND (González-Herrero et al., 2022).

Gender dysphoria can result in altered functioning in the sensorimotor and attention circuitry (Lin et al., 2014), possibly through alteration of the neurological body map (Walsh & Einstein, 2020). Through this FND could emerge as a coping mechanism in gender dysphoria, and altered sensorimotor and attention circuitry functioning can potentially increase the risk of developing FND (Drane et al., 2020). Another explanation is that gender dysphoria could result in increased somatic ruminations, meaning that psychological distress was more likely to manifest in physical symptoms.

It is possible that there is a hormonal contributor to the sex and gender differences seen in FND. Testosterone and estradiol exposure affect the likelihood of presenting with externalizing and internalizing disorders, respectively (Martel, 2013). It is unclear if this is secondary to the direct effect of the hormone in the brain or due to the effects of fluctuating estrogen levels. The possible role of sex hormones in the pathogenesis of FND specifically is evidenced by the gender differences being greatly altered by puberty and menopause, the former increasing the incidence in people assigned female at birth and the latter reducing it (Asadi-Pooya & Homayoun, 2020; Baizabal‐Carvallo & Jankovic, 2020; Campo et al., 1999).

Given that human stress responses are mediated by sex hormones, oxytocin, and endogenous opioids *via* the modulation of sympathetic and hypothalamic-pituitary-adrenal axis activity, it is reasonable to postulate that estrogen and progesterone may play a role in the pathogenesis of FND (Chouksey & Pandey, 2020). Evidence for testosterone having a treatment effect on somatic symptoms is that testosterone GAHT has previously been found to treat fibromyalgia in trans men, but estradiol GAHT exacerbated symptoms in trans women (Aloisi et al., 2007). Although the authors of the trial concluded that testosterone and estradiol alter pain perception, it is possible that this effect was due to their respective propensity to form somatic symptoms.

The potential role of sex hormones in the pathogenesis of FND means that FND may be able to be treated with hormone therapy in trans and cisgender people (only in gender affirming manner). It also could mean that the specific dosing, timing, and formulation of GAHT could be highly relevant to the psychiatric outcomes of GAHT. Unfortunately, this level of information has not been reported in previous studies.

### Limitations

There are several reasons to be cautious in drawing conclusions from this study, most obviously the small number of studies reviewed. Some articles did not label the condition affecting their participants as FND. The two case reports/series that did not label the condition FND described symptoms consistent with FND and were thus included. Whilst gender affirmation improves mental health, it does not remove the widespread societal discrimination and lack of acceptance more broadly of trans people, so it may be optimistic to expect an improvement in FND symptoms with gender affirmation alone. While the physical changes from GAHT can bring happiness and relief of dysphoria, they can increase social challenges born out of stigma and discrimination against trans people. What’s more, the relationship between the resolution of FND and improvement in general mental health and wellbeing may not be causal. It is possible that there was reporting bias in the cases, as cases in which social affirmation or GAHT appeared to remedy FND symptoms may be more likely to be published. Finally, the studies did not aim to examine the sex or gender differences in FND and there may be missing or unreported relevant information.

There may be an understandable sensitivity to the suggestion that FND may have a biological explanation, given the historical medical stigmatization of cisgender women with FND (McLoughlin et al., 2023). However, if there is a hormonal role in its pathogenesis it may have implications for treatment and deserves to be investigated. Likewise, there may be sensitivity to the suggestion that FND has a psychiatric explanation, due to the historical attribution of physical symptoms of cisgender women to psychopathologic etiologies. There may also be understandable sensitivity to the studying of the possible biological effects of GAHT, given psychiatry’s historical pathologization of trans people. This review found evidence of continued stigmatization of trans people in some areas of the medical community, with some of the articles included using pathologizing language, which was also present in the search terms of some databases.

### Conclusion

This review examined the literature on trans people with FND. It found that trans people were likely overrepresented in FND and somatic symptom and related disorders were slightly overrepresented in the trans population. It is the first systematic review of FND in trans people to challenge the traditional concepts of female predominance seen in cisgender people by highlighting that that gender ratio does not account for trans people, and this review provides hypotheses regarding the role of sex hormones in FND. Other reviews on the subject have focused solely on the unique experiences and care needs of trans people with FND and have been less complete than this review, in terms of the studies included (Boerner et al., 2023; Lerario et al., 2023; Lerario et al., 2023). We found that gender affirming care was associated with an improvement in symptoms of FND, however there was insufficient evidence as to what aspect of gender affirmation affected FND symptoms. We also found that trans people make up a not insubstantial portion of people with FND. More research is needed as to why this is the case and how medical providers can better cater for their treatment needs. Examining the course of FND in trans people may not only shine a light on the unique challenges facing trans people with FND, but also help further understand the sex and gender differences seen in FND, and possibly pave the way for future research. The finding that trans people may be overrepresented in the FND population could potentially affect clinician biases, as occurred in Williams (2012), and care must be taken to assess for biases in clinical practice and use inclusive and appropriate language. Future adequately powered studies with data gathered expressly for the study could be illuminating.

## Supplementary Material

Supplemental material.docx

## Registration

This systematic review was registered and can be accessed on Prospero (ID CRD42024517386).
