# Supplementary material for: A systematic review of functional neurological disorder in transgender people
Source: Int J Transgend Health. 2025 Jul 25;27(5):2753–65. doi: 10.1080/26895269.2025.2538742 (PMC13618163; doi:10.1080/26895269.2025.2538742)
Supplement: Supplementary file 1 [file WIJT_A_2538742_SM3978.docx]

| Table 1: Search Strategy | | | |
| --- | --- | --- | --- |
| Concepts (combined with “and”) | OVID (MEDLINE) | PsycINFO | Web of Science |
| Functional neurological disorders | “somatoform disorders” | “somatoform disorders” | “conversion disorder” or “conversion disorders” or “dissociative seizures” or “functional neurological disorder” or “functional neurological disorders” or “functional neurological symptom disorder” or “functional neurological symptoms” or “functional movement disorder” or “functional movement disorders” or somatoform disorders” or “nonepileptic seizures” or “PNES” or “pseudoseizures” or “psychogenic” or “psychogenic movement disorder” or “psychogenic seizures” or “psychogenic non-epileptic seizures” or Psychogenic nonepileptic seizures” |
| Trans/gender diverse | “sexual and gender minorities” | “gender identity” or “gender identity disorder” | “sexual and gender minorities” or “transgender persons” or “gender-nonconforming persons” or “gender dysphoria” or “gender identity” or “gender identity disorder” or “transgender” or “gender diverse” |
| The searches were conducted on 08/04/2024. A secondary search was conducted on Google Scholar and the bibliographies of papers were also manually searched for relevant papers. | | | |
